# Effects of sex hormones on survival of peritoneal mesothelioma

**DOI:** 10.1186/s12957-015-0624-4

**Published:** 2015-06-26

**Authors:** Yeqian Huang, Nayef A. Alzahrani, Winston Liauw, David L. Morris

**Affiliations:** St George Clinical School, University of New South Wales, Sydney, New South Wales Australia; College of Medicine, Imam Muhammad ibn Saud Islamic University, Riyadh, Saudi Arabia; Department of Medical Oncology, St George Hospital, University of New South Wales, Sydney, New South Wales Australia; Hepatobiliary and Surgical Oncology Unit, Department of Surgery, St George Hospital, University of New South Wales, Level 3 Pitney Building, Gray Street, Kogarah, Sydney, New South Wales 2217 Australia

**Keywords:** Peritoneal mesothelioma, Hormone, Female, Survival

## Abstract

**Background:**

Previous studies have suggested the presence of steroid receptors as a favourable prognostic factor in peritoneal mesothelioma (PM). This study aims to investigate possible hormonal effects on survival of PM.

**Methods:**

This is a retrospective study of prospectively collected data of 52 consecutive patients with PM who underwent cytoreductive surgery (CRS) and hyperthermic intraperitoneal chemotherapy (HIPEC) by the same surgical team at St George Hospital in Sydney, Australia, between April 1996 and April 2013. Females were arbitrarily divided into assumed premenopausal (<51 years old; *n* = 15) and assumed postmenopausal (≥51 years old, *n* = 9). In each gender group, patients were furthered divided into three age groups (<40, 40–60, >60). A significant statistical difference was defined as *p* < 0.05.

**Results:**

Females with epithelial mesothelioma had a significantly higher survival than males (*p* = 0.023). They also had a better overall median survival (>60 months) than males (43 months), although this difference was not statistically significant (*p* = 0.098). Survival of postmenopausal females became similar to males after excluding benign cystic mesothelioma.

**Conclusions:**

The better survival in premenopausal females could probably be explained by higher levels of oestradiol and progesterone. Also, our data suggests that higher rates of benign cystic mesothelioma in females was not the key reason for the better survival in female patients, further supporting the hypothesis of hormonal links with survival of PM. Therapeutic effects of sex steroid hormones on PM may be a valuable area to explore.

## Background

Mesothelioma arises from serosal lining of the pleura, peritoneum, pericardium or tunica vaginalis [1]. Histological appearance of mesothelioma ranges from a well-differentiated cystic variant to a poorly differentiated sarcomatoid variant [2]. The peritoneum is the second most common site for mesothelioma, contributing one third of all cases [3]. Common presentations of peritoneal mesothelioma (PM) include increased abdominal girth, abdominal pain, abdominal mass or ascites and weight loss. Due to nonspecific symptoms, diagnosis is often delayed [3, 4]. Systemic chemotherapy has shown to have limited efficacy [5]. The current approach for mesothelioma is a combined locoregional treatment which consists of cytoreductive surgery (CRS) and hyperthermic intraperitoneal chemotherapy (HIPEC) for suitable patients. This approach has been shown to prolong the survival of PM and achieve a median survival of up to 60 months [6].

There is a clear relationship between asbestos exposure and mesothelioma. Higher incidence of asbestos-related occupations among males has been suggested to account for the higher risk of mesothelioma in males. Nevertheless, mesothelioma also occurs in 20 % of patients without previous clearly identified asbestos exposure, suggesting that other factors may be responsible for the pathogenesis in this disease [7]. Female patients have been consistently reported to have a better prognosis than male patients in previous studies [4, 6, 8–11]. The prognostic difference between sexes has suggested a possible hormonal link with the survival of this disease. Also, a few histological studies have demonstrated the presence of sex steroid receptors in PM [3, 7, 12, 13]. Thus, the aim of this study was to assess hormonal effects on survival of PM.

## Methods

### Settings

This is a retrospective study of prospectively collected data of 52 consecutive patients with PM who underwent CRS and HIPEC by the same surgical team at St George Hospital in Sydney, Australia, between April 1996 and April 2013. All the clinical and treatment-related data were collected and entered into a computerised database in order to evaluate the survival outcomes of patients with peritoneal mesothelioma. A signed informed consent to use their clinical data for research purposes was obtained from all patients prior to their surgery. This study was ethically approved by South Eastern Sydney Local Health District Human Research Ethics Committee, New South Wales, Australia.

### Patients

Patients had a good performance status (World Health Organization Performance Status ≤2) and had a histological diagnosis of peritoneal mesothelioma. Histological diagnoses in this study included benign cystic mesothelioma, epithelial mesothelioma, sarcomatoid mesothelioma and biphasic mesothelioma (mixed epithelial and sarcomatoid variants). All patients were managed by a standard treatment protocol which includes CRS and HIPEC. Suitability to undergo CRS and perioperative intraperitoneal chemotherapy (PIC) was evaluated during a regular weekly meeting attended by a multidisciplinary team including surgical oncologists, medical oncologists, radiologists, cancer care nurses and research staff. Survival differences between sexes were analysed. All patients were then further divided into three groups for survival analysis including premenopausal females, postmenopausal females and males. Premenopause was defined as less than 51 years old (*n* = 15) whereas postmenopause was defined as at least 51 years old (*n* = 9). Subgroup analysis compared survival outcomes of different mesothelioma subtypes within each gender group. Also, survival of three age groups (i.e. aged less than 40, between 40 and 60 and greater than 60 years old) was also compared within each gender group (Females: <40 group *n* = 7; 40–60 group *n* = 14; >60 group *n* = 3; Males: <40 group *n* = 5; 40–60 group *n* = 16; >60 group *n* = 7).

### Preoperative management

All patients underwent standard preoperative investigations which included physical examination, double contrast-enhanced computed tomography scans of the chest, abdomen and pelvis, as well as positron emission tomography.

### CRS

A standardised treatment protocol combining CRS and PIC was performed by the surgical team. An initial assessment of the volume and extent of disease was recorded using the peritoneal cancer index (PCI), as described by Jacquet and Sugarbaker [14]. This assessment combines thickness of lesion size (LS) (LS 0: no macroscopic tumour; LS 1: tumour <0.5 cm; LS 2: tumour 0.5–5 cm; and LS 3: tumour >5 cm) with tumour distribution (abdominopelvic region 0–12) to quantify the extent of disease as a numerical score (PCI 0–39). The aim of CRS was to remove all macroscopic intraperitoneal and visceral tumour deposits. CRS was performed using Sugarbaker’s technique [15].

All sites and volumes of residual disease following CRS were recorded prospectively using the completeness of cytoreductive (CC) score (CC0—no macroscopic residual cancer remained; CC1—no nodule >2.5 mm in diameter remained; CC2—nodules between 2.5 mm and 2.5 cm in diameter remained; CC3—nodules >2.5 cm in diameter remained) [14].

### HIPEC

After CRS, but prior to intestinal anastomosis or repair of seromuscular tears, HIPEC was performed by installation of a heated chemoperfusate into the abdomen using the coliseum technique. Cisplatin (100 mg/m^2^ in 1000 ml normal saline) and mitomycin (12.5 mg/m^2^ in 1000 ml normal saline) were given simultaneously over 90 min.

### Statistical analysis

All statistical analyses were performed using IBM SPSS for Windows version 22. Comparison of normally distributed variables was performed using analysis of variance (one-way ANOVA) test. Categorical variables were analysed using chi-square test or Fisher’ exact test where appropriate. Median survival was calculated based on the last time of contact or death in the unit of months. Survival analysis was performed using the Kaplan-Meier curves and log-rank test for comparison. A significant difference was defined as *p* value less than 0.05.

## Results

### Descriptive characteristics

Table 1 demonstrates the background characteristics of our patients with mesothelioma. There was a significant statistical significance in terms of histological diagnosis (*p* = 0.045). Of females, 29.1 % were diagnosed with the benign cystic form of mesothelioma as compared to 3.7 % in males. More males (18.5 %) were diagnosed with malignant biphasic mesothelioma as compared to 8.3 % in females. There was no statistical difference in terms of age, mean PCI, CC score, transfusion units and HIPEC between females and males.

**Table 1:** Background characteristics

	Female	Male	
Total *n* = 52			*p*
*N* (%)	24 (46.2)	28 (53.8)	
Age mean (SD)	46.8 (49.5)	51.8 (50.0)	0.147
Diagnosis *n* (%)			0.045
Benign cystic	7 (29.1)	1 (3.7)	
Epithelial	14 (58.3)	21 (77.8)	
Sarcomatoid	1 (4.2)	0 (0)	
Biphasic	2 (8.3)	5 (18.5)	
PCI mean (SD)	17	22	0.099
CC *n* (%)			0.117
0	17 (73.9)	13 (46.4)	
1	6 (26.1)	14 (50.0)	
2	0	1 (3.6)	
3	0	0	
Transfusion mean (SD)	4.75 (4.54)	7.21 (7.81)	0.180
HIPEC *n* (%)			0.872
Yes	22 (91.7)	26 (92.9)	
No	2 (7.7)	2 (7.1)	

### Survival analysis—epithelial type of mesothelioma

**Fig. 1:**
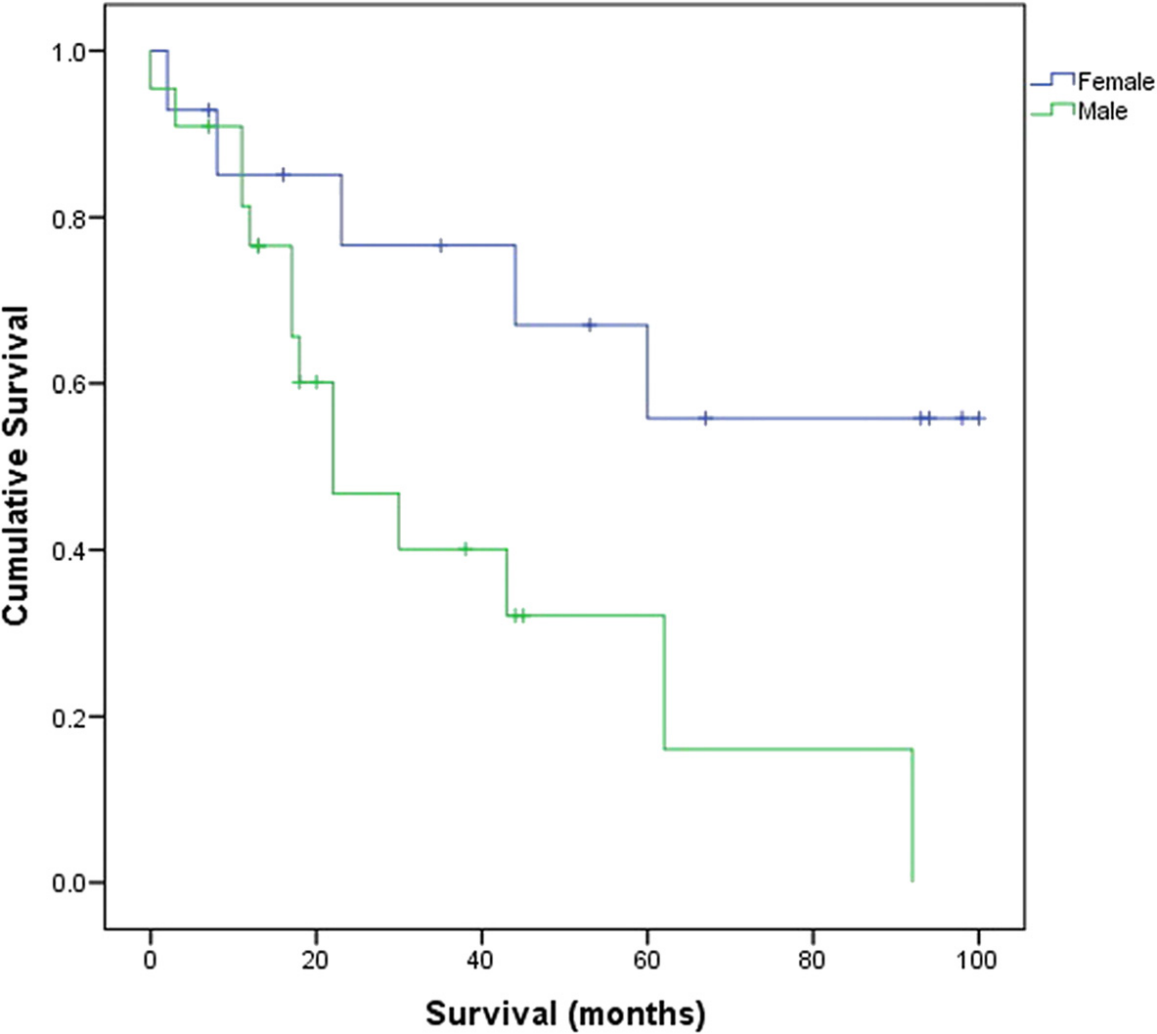
Survival curve for epithelial mesothelioma

Due to the small numbers of patients with sarcoma and mixed variants, the statistical analysis of all histological subtypes could not be performed. However, survival was significantly higher in females with epithelial type of mesothelioma (*p* = 0.023). As half of the female patients with epithelial mesothelioma were still alive at the time of analysis, median survival could not be calculated for female patients. However, according to the survival curve, the median survival in females with epithelial type of mesothelioma was greater than 60 months as compared to survival in males (median = 22.0 months, 95 % confidence interval (CI) = 7.9–36.1) (Fig. 1)

### Survival analysis—females vs. males

**Fig. 2:**
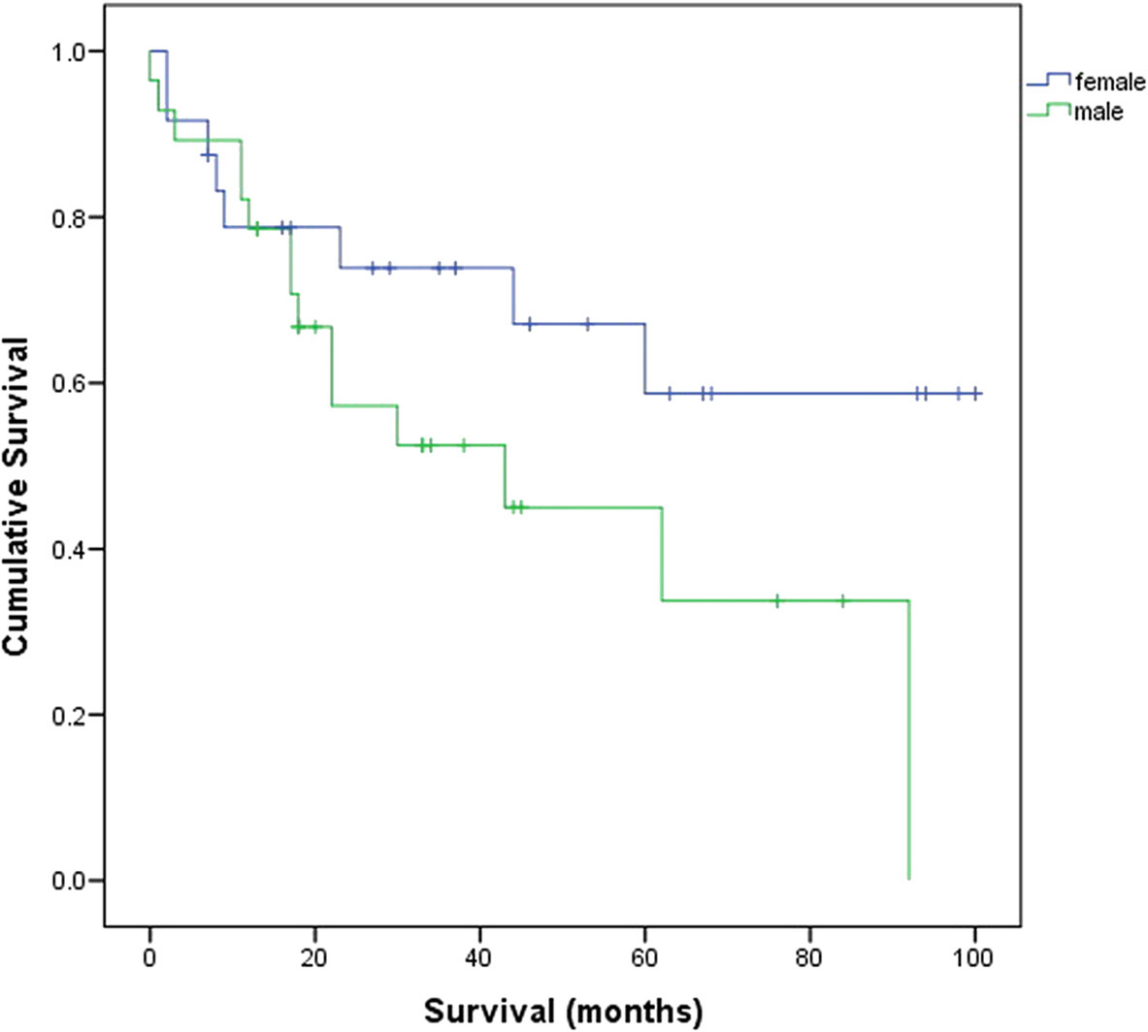
Survival curve for males and females

As more than 50 % of patients remained alive at the end of this study, median survival of all females, premenopausal and postmenopausal females was unable to be calculated. It was shown that females had a better median survival (>60 months as shown in Fig. 2) than males (median = 43.0 months, 95 % CI = 5.5–80.5), although this difference did not reach a statistical significance (*p* = 0.098).

### Survival analysis—premenopausal females vs. postmenopausal females vs. males

**Fig. 3:**
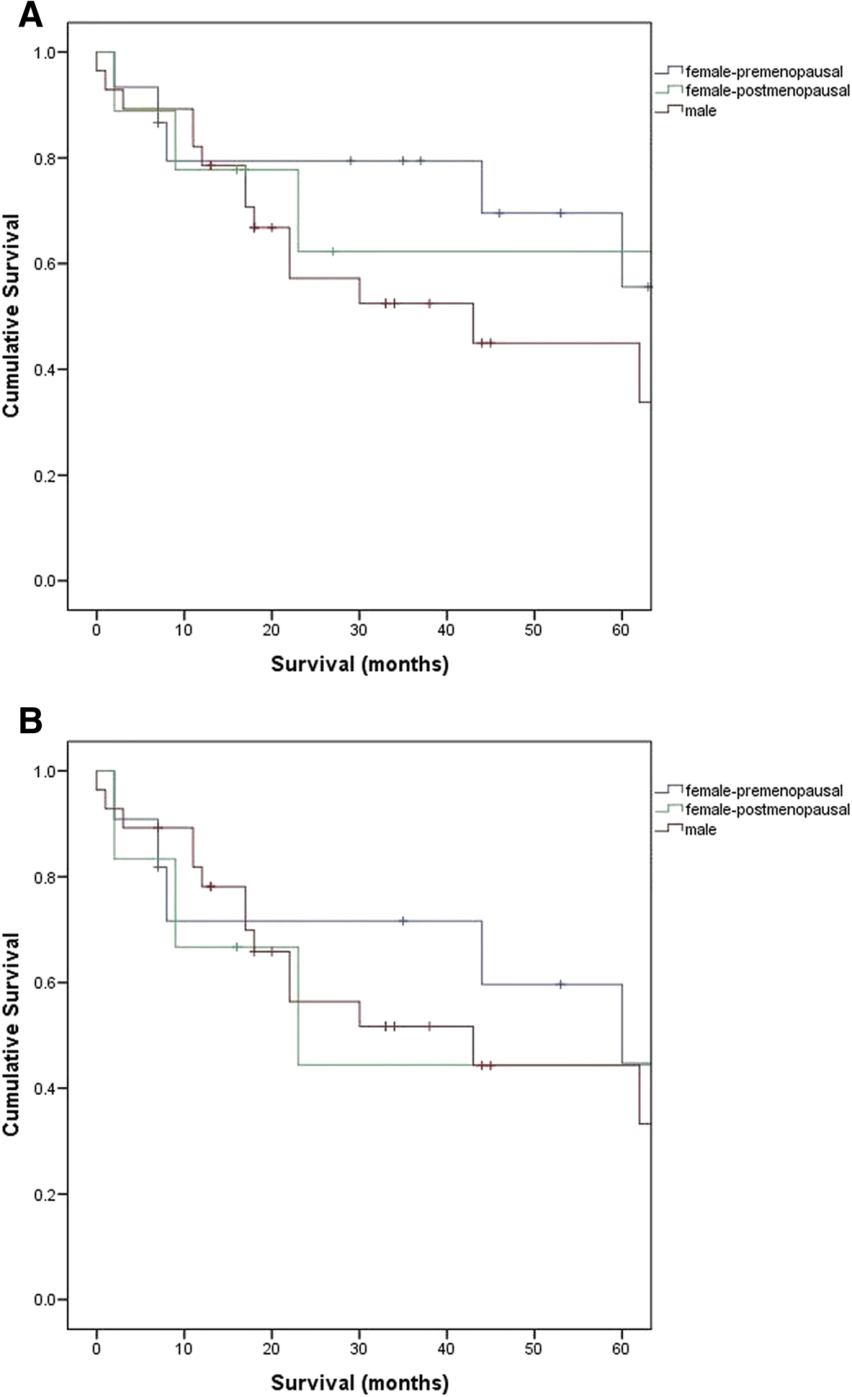
Survival—premenopausal females vs. postmenopausal females vs. males (**a** includes benign cystic mesothelioma; **b** excludes benign cystic mesothelioma)

Figure 3 compares the survival curve including benign cystic mesothelioma with the curve excluding benign cystic mesothelioma. Two survival curves showed similar trends in all three groups. However, survival of postmenopausal females became more similar to males after excluding benign mesothelioma. It also showed that premenopausal females have better survival then the other two groups. However, there was no statistical significance in terms of median survival among premenopausal females, postmenopausal females and males (*p* = 0.253).

### Subgroup survival analysis: <40 vs. 40–60 vs. >60

**Fig. 4:**
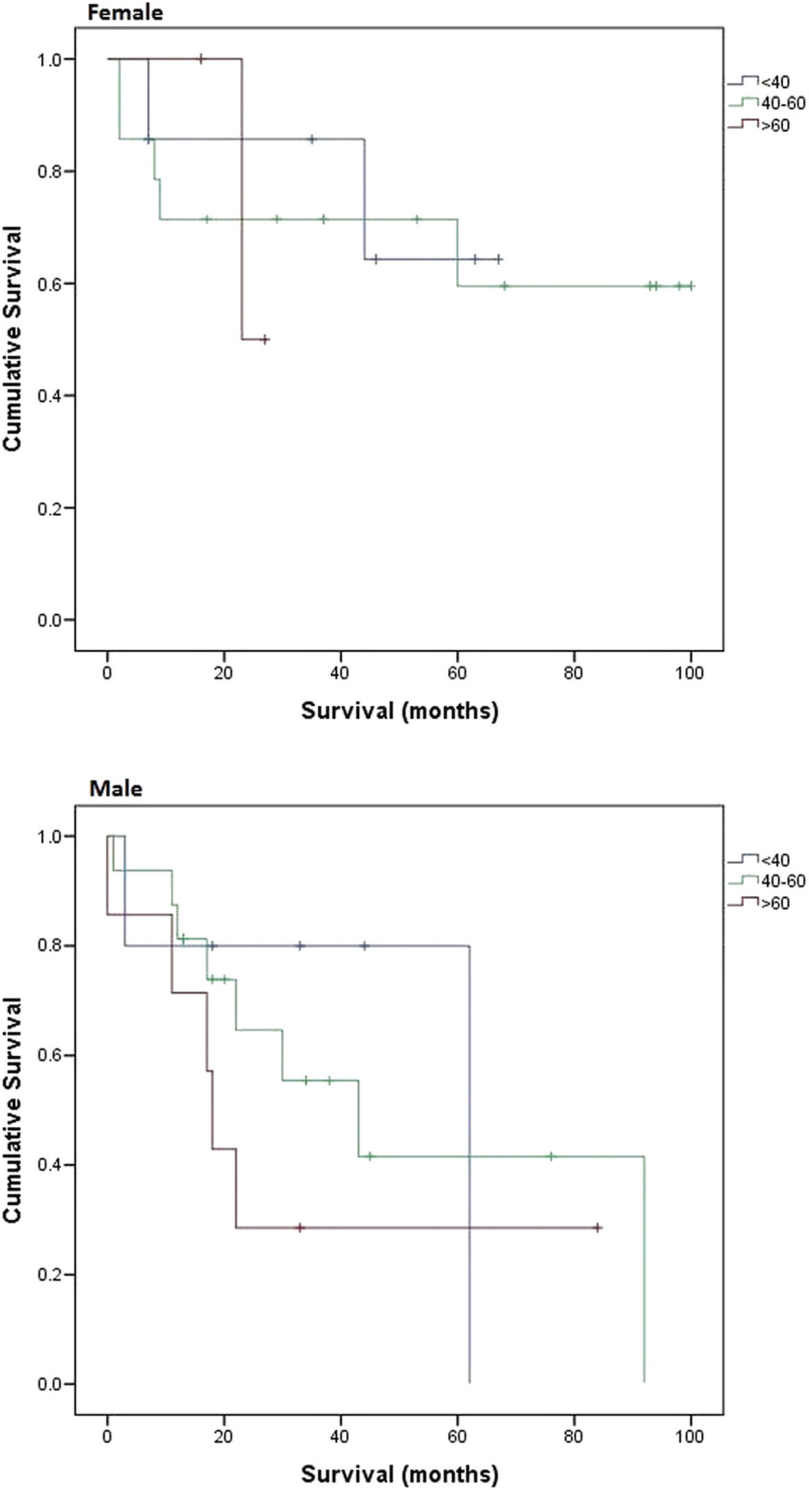
Survival subgroup: <40 vs. 40–60 vs. >60

Since more than 50 % of patients remained alive at the end of this study, median survival of females aged <40 and females aged between 40 and 60 was unable to be calculated. The median survival of females aged >60 was 23 months (95 % CI could not be calculated). The survival curve showed that patients aged ≤60 have a better median survival than those aged greater than 60 (Fig. 4). However, such a difference did not reach a statistical significance (*p* = 0.952).

In the male group, median survival of those aged less than 40, between 40 and 60 and greater than 60 was 62 months (95 % CI could not be calculated), 43 months (95 % CI = 13.5–72.5) and 18 months (95 % CI = 15.4–20.6), respectively. Although the difference was not statistically significant (*p* = 0.486), the survival graph showed a trend of improving survival in younger male patients (Fig. 4).

## Discussion

Mesothelioma is aggressive and fatal. The differential expression of hormonal receptors has been suggested to be correlated with survival differences between sexes in PM [3]. A recent study done by our group, Pillai et al. [7], investigated the role of oestrogen receptors in malignant PM (MPM). They identified nuclear oestrogen receptor-β (ER-β (*n*)) as a favourable prognostic factor in PM and suggested that high level of oestradiol may explain the survival difference in sexes, since ER-β (*n*) is oestradiol-dependent [7]. Also, the study done by Horita et al. suggested that progesterone induces apoptosis in malignant mesothelioma cells. Our data is consistent with these hormonal findings. We have seen a significantly better survival in females with the epithelial type in this study. Better survival outcomes in assumed premenopausal females and similar survival trends between postmenopausal females and males shown in our study could be explained by a high level of oestradiol and progesterone in premenopausal patients (Fig. 3). Although we have not measured sex hormones or their receptors in this study, our finding is consistent with previous literature in pleural mesothelioma [16, 17]. The study done by Taioli et al. reviewed 14,228 patients with MPM and showed less apparent difference in survival for females over age 50 years. Wolf et al. studied 715 cases of patients with MPM and showed that young females are disproportionately represented among long-term survivors of MPM [18]. They suggested that hormonal status may contribute to better survival in female patients with MPM.

Our subgroup analysis findings suggest that females have a better survival in the epithelial mesothelioma group as compared with males. Given that epithelial variant was suggested to be a favourable prognostic factor, such a significant difference may further suggest the key role of sex steroids and/or hormonal receptors in the survival of PM. In addition, our findings also show that younger patients have better survival in both gender groups. This could be contributed by a high level of oestradiol in females. Also, free and bioavailable oestradiol levels decline significantly with ageing in males [19]. This could contribute to the much higher survival in male patients aged <40 as compared with those aged >60 (62 vs. 18 months, respectively). However, the evidence to support these possible explanations is insufficient from our data.

Overall survival was higher in females with PM than males, which is consistent with previous studies [3, 5, 7–10]. A higher incidence of MPM in males was also demonstrated. Nevertheless, our data suggests that higher rate of benign cystic mesothelioma in females was not the key reason for greater survival in female patients given similar survival curves shown in Fig. 3, supporting the hypothesis of a hormonal link with the survival of PM.

Our study is the one of the few clinical studies that evaluated the link between sex steroid hormones and survival of patients with PM. However, this is a retrospective study conducted at a single centre. Given that suitability of patients for peritonectomy was discussed during weekly meetings and only selected patients were offered this combined therapy, there was selection bias in this study. Also, our study was limited by the small sample size due to the low incidence of this disease. A similar study design could potentially be applied for a greater population in order to further explore the link between sex steroid hormones and survival of PM.

## Conclusions

In conclusion, oestrogen and progesterone may prolong the survival of patients with PM. Therapeutic effects of sex steroid hormones on PM may be a valuable area to explore. Potential topics for future research include the survival outcomes of young females with and without hysterectomy, as well as postmenopausal females on hormonal replacement therapy.
